# General Evidence on Behalf of Mrs. Cumming

**Published:** 1852-04-01

**Authors:** 


### GENERAL EVIDENCE ON BEHALF OF MRS. CUMMING.

*Mr. Francis Farrar*, examined.—Is at present the managing-clerk in the office of Messrs. Stokes and Hollingsworth. In May, 1835, was in practice as a solicitor: succeeded to Mr. Freame, who had been previously concerned for Mrs. Cumming. When witness first knew Mrs. Cumming, she was living in Ebury-street, Pimlico, in an apparently highly respectable manner. During 1835 and 1836 was in frequent communication with Mrs. Cumming. In 1836 I went to Newport to collect her rents. The property was settled upon Mrs. Cumming for her separate use. I observed nothing particular about the state of her mind. She gave me instructions quite as a woman of business. In 1838 I was at Newport with Mrs. Cumming. She examined the state of the property, conversed with the different tenants as to the repairs, and gave directions as to what she should allow in the shape of material, they providing the labour. At that time I remember Miss Thomasine Cumming—she had married Mr. Hooper. Mrs. Cumming had spoken to me about the marriage, and said that she considered Mr. and Mrs. Ince encouraged Mr. Hooper's attentions. She requested me to go and see Mr. and Mrs. Ince on the subject, and I did so; that was before the marriage. I reported Mrs. Cumming's great objection. Mrs. Ince stated that they had no power to dissuade Miss Cumming, if she chose to make the connexion: at any rate they would do their utmost, as it was a most objectionable match. In consequence of that, Miss Cumming, who had previously been at the Inces', returned home. Mrs. Cumming afterwards told me that after she had returned home Mrs. Ince had allowed her to be married from her house. Mrs. Cumming expressed her feeling that Mrs. Ince had acted most improperly and unkindly towards her sister. She was a person who sometimes expressed herself strongly. She was an irritable person. She expressed herself strongly on this occasion. My acquaintance as a professional man continued to the beginning of 1843; subsequently it was not so constant. I knew Captain Cumming also; he was a very irritable person also. I have heard him using very violent language to his wife, cursing and swearing. He took the benefit of the Act in 1839. I remember some of her furniture being seized for his debts.

She had moved part of it away for protection. Mrs. Cumming told me she had given Mr. Ince money to buy in a silver basket, sometime before Capt. Cumming took the benefit of the act. She stated, that although it was purchased she never could get the basket back—at least she did not get it back for a long while. She was very much annoyed indeed. She had asked for it repeatedly, both from Mr. and Mrs. Ince. She said they had stolen it; that they had kept the money and stolen the basket. She gave me the notion of being a person imperfectly educated. Besides the debts of her husband, and the furniture being seized, she complained of the irregularities of her husband in his amours. The first time was when he was confined in the Queen's Bench (1840?), when she stated to me that his servant had represented Mr. Cumming's conduct as very indecent to her. The servant subsequently repeated it to me herself.

Sir F. Thesiger.—Never mind what the servant said. — I repeated to Mrs. Cumming what the servant had said. The servant stated to me that Mr. Cumming had endeavoured to take improper liberties with her. and she knew that he had repeatedly improper women in his room with him. The Captain was in prison nearly two years. When he came out he went back to reside with Mrs. Cumming. In the year 1842, she told me that he was continuing similar conduct with the female servant, and that in consequence of her refusing to discharge this servant, he refused to eat or drink anything. She requested me to go up and talk to him. I reasoned with him upon the absurdity of his conduct. He admitted it, and subsequently had a tray up and ate some food. I never observed that she kept him short of provisions; on the contrary, when in the Bench I saw her take him various delicacies, give him money, and expressed to this very servant I have been speaking of, her great desire that every comfort and attention should be paid to him. I have dined with them. I remember her having some cats; three or four, certainly not more. I saw nothing extraordinary about her treatment of them. I have seen them in different parts of the house. I never saw one in her lap. Shortly before she was taken to York House in 1846, she called on me and stated that he (Captain Cumming) had been very violent, and had scratched her hands in endeavouring to remove her rings from her, and had taken her rings away. She showed me her hands. I saw there were scratches—trifling scratches. She told me that he had returned the rings. She told me that he had pawned her watch. I saw her not more than a fortnight before her removal to York House, on a matter of business relative to a notice from some Water Works Company that was about to take some property of hers at Newport. I was not concerned for her then. She showed me the notice which had been served upon her. She asked me what I thought she had better do about it. I do not remember who was acting for her at that time. I was at Messrs. Stokes and Hollingsworth's. I had some conversation with her about investing the money if she got it. She talked on that occasion coherently and rationally. I saw nothing about her manner leading me to believe her irrational or of unsound mind. I received a note from her requesting me to visit her in York House. I went and saw her. I was accompanied by a brother, a medical man, who is now abroad. She described the manner in which she had been removed: that upon some one knocking at the door, she desired the servant to look out of the window and see who it was. The servant told her there were two or three people desirous of seeing her; that she went down into the hall to speak to them through the door, for that she would not have it opened to them, but that the servant partially opened the door, and that they then pushed themselves in. After they had come in she spoke to them for some minutes in the hall herself; that then a female stated it was of no use what she said, she must go with them, and that if she did not do so quietly, she must put on her a strait waistcoat, taking one from under her shawl at the same time. I think she told me that it was put on; at any rate, that they forcibly removed her from the house, and she screaming murder at the time; that they put a shawl over her head to prevent her cries being heard, and pushed her into a carriage in waiting—not on the seat, but into the hollow part where the feet go, and then drove on; that she was without bonnet, and, in fact, without any proper clothing to go out of doors in.

(At this period Mr. James intimated his wish that Mr. Ebenezer Jones should leave the room, saying that he had been communicating with all the witnesses. Sir F. Thesiger, addressing Mr. Jones, said he had been repeatedly told to leave the room.)

The witness continued:—She told me she had endeavoured to escape from the madhouse. She wished me to ascertain by whose means she was put there. She might then have been about a fortnight in the asylum. She told me she had requested a lady who went to church to post a letter to me. She wished me to adopt every means in my power to get her released. While I was present Sir Alexander Morison called and examined her. After that I saw Lord Ashley and several of the Commissioners. I went to see Mrs. Cumming a second time. I introduced to her my brother, who is a solicitor in Doctors' Commons. I asked the matron to let me see the certificate under which she was detained. She refused to show it to me. I could not get the certificate from the Commissioners. I went to Captain Cumming.

Sir F. Thesiger here objected that what passed between the witness and Captain Cumming was not evidence. Mr. James submitted that it was; it had been prominently brought forward as one of the reasons why Mrs. Cumming should not entertain an aversion to her children that the proceeding originated with Captain Cumming, and not with her daughters; he (Mr. James) was in the course of proving that it did not originate with Captain Cumming, and that it did originate with them. Of course he must abide by the judgment of the Commissioner, but if he rejected that evidence, he must ask him to take a note that it was offered. Sir F. Thesiger said that would be futile, because there was no appeal; there could be nothing like an application for a new trial. Mr. James submitted that if evidence were improperly received he should move the Lord Chancellor to quash the inquisition. The Commissioner considered his taking a note of the objection as mere "waste paper." Ultimately, Sir F. Thesiger having stated that Captain Cumming had signed the order, Mr. James contended that if what Cumming wrote was evidence, what he said was evidence; and the Commissioner being informed by the witness that he considered his brother, who accompanied him to Captain Cumming, as her solicitor, admitted the question.

Q. Now, then, in the course of the investigation as to the circumstances how she became confined there, and through whom, what did you say to Captain Cumming? I asked him if he knew where Mrs. Cumming, his wife, was? He said he did not. I asked him if he knew through whose authority she had been taken to York House. He was a very deaf man. I desired the nurse to repeat it. She did so audibly. He said he knew nothing at all about it. I went to Mr. Ince. He refused to see me. He referred me to Mr. Dangerfield, his solicitor, the same who has been examined here as a witness. I did not see Mr. Hooper. I went to Mr. Dangerfield. I asked him by whose authority Mrs. Cumming was placed at York House. He said, "You must put your application in writing, and you shall have an answer." My brother wrote. We got an answer refusing any information. I was opposed access to Mrs. Cumming again. I did not know anything at all about the inquiry (at the Horns Tavern) till the morning of the second day. Being refused admission to her at the asylum, I was quite unable to procure any defence for her. I saw her at the investigation, and conversed with her. — I believe I have stated it correctly, because I have stated it with the knowledge of the Commissioner, that she appeared there at that commission undefended altogether,—the whole of the first day. I believe she told me so herself. — And was I right in stating to the jury that Mr. Haynes was there accidentally, and she consulted him upon the subject? She did.

Cross-examined.—I have not my bill of costs; when I last saw it, it was in Mr. Dangerfield's possession. — Was not Mr. Ince desirous that Mrs. Cumming should interpose if she wished to prevent the match (Mrs. Hooper's)? I think it very likely he was; I have no doubt of it. I have not the least recollection whether I heard from Mrs. Cumming that Mr. Ince had taken various means to induce her to interfere. I do not know that Mr. Ince pointed out any specific mode in which she was to interfere. I have no doubt he wished her to interfere. Mrs. Cumming did interfere. I acted as Mrs. Cumming's solicitor for about seven or eight years. The rental at that time was nearly 600*l.* a year. When I went down to Newport the Red House was very much dilapidated. There was a lease upon it, upon the footing of its being repaired by the person who took the lease. It was worth a great deal more after the repairs were done. There was one property near the sea-wall, the house was in a very dilapidated state; with that exception, and the Red House, the rest was in tolerable repair. That was in 1836, and then in 1838. I think Captain Cumming was in prison in 1837, and discharged in 1839. — Have you heard from Mrs. Cumming that the detaining creditor was the person who was the grantee of an annuity charged on the Red House? Yes. — Did you not hear from Mrs. Cumming that her husband was merely a security in respect of that annuity? No; I should have said quite the contrary, that she was the security. — Was it not by reason of the non-repair of the Red House that the annuity became so in arrear? I believe so. — And that compelled the creditors to sue Captain Cumming for arrears of this annuity, which led to this insolvency? I think he would have sued Captain Cumming long before if he had known where to find him. Mrs. Cumming had a great dread of letting this house, it would be the means of giving the party entitled to the annuity the address of herself and her husband. I think the annuity had not been paid for many years. With the exception of giving evidence I did not assist at all at the last commission. I have had conversations with Mr. Haynes upon the subject of the arrangement entered into. I have not assisted at all in procuring witnesses upon this occasion. It was in 1842 that the conversation took place upon the subject of Captain Cumming. He was then eighty years of age. He told me so himself. He was not in strong health. I saw no peculiar feebleness about him. He drank his wine very freely. It was accidentally that I observed the scenes of violence between Captain and Mrs. Cumming. It was as many as four times.

Re-examined.—These scenes occurred more than once when I have called on business. — About the detaining creditor. Do you know what the amount of Captain Cumming's debts were? She has told me he owed thousands of pounds. His address was never known. It was thought that she was merely tenant for life. I know that people who are tenants for life are not so particular as others. She must have been sixty odd at that time. The houses were all tenanted, except the Red House, and at pretty good rents. The Red House is the one that was afterwards taken for improvements. — Did Mrs. Cumming complain after the girl (Miss Cumming) returned home, and Mr. Ince had acceded to your remonstrance, that she was married from Mr. Ince's house? She complained of it.

By Sir Frederick Thesiger.—I did not know that I was a legatee in a will of Mrs. Cumming's, until I heard you read something of the kind the other day. Mr. Haynes had asked me, when the matter was before the Lords Justices, if I did not know that my name was in the paper. He did not tell me the amount.

Re-examined.—I had endeavoured to procure Mrs. Cumming's release from the asylum. I did not act as her attorney, or make any charge. She has expressed the most kindly feelings to me.

By the Commissioner.—As to the house near the sea-wall, there was some notion of that coming down, and two farms being thrown into one. When I saw Mrs. Cumming in the asylum, she requested me to bring my brother to her.

By a Juryman.—I do not know that she kept any account or memorandum of the rents that she received.

By the Commissioner.—I went down to collect the rents in 1836. She brought me the receipts written by herself. It was the only intimation I had of who the tenants were; she gave me a list of them.

*Mr. Edward Henry Hawkins*, examined.—Am assistant-clerk to a provision-merchant—formerly resided at Newport—received Mrs. Cumming's rents from 1839 to 1844. She herself attended to the general management of her property. She gave me the receipts for the rents, and corresponded with me upon business matters. I received the following letter from Mrs. Cumming, in her handwriting:—

"June 30th, 1843.

"Dear Sir,—I should feel much obliged by your sending me a statement of the various remittances of the last August rents, stating by what bankers it was sent to London, and the amount of each remittance. You will, I doubt not, receive the rents as usual from the tenants, and also send me up the stamp receipts for the August rents for me to sign; and you will have the goodness to inform the tenants at the same time I shall expect my rents when due. Requesting an early answer, I remain, Yours obediently,

"Catherine Cumming."

The witness produced other letters from Mrs. Cumming.

"London, 15th December, 1843.

"Dear Sir,—Enclosed I send you four receipts out of the five, keeping back William Morgan's, as I shall take the rent. William Morgan, through having underlet the house, forfeited his lease. I shall take up the house. My eyes still continue so very bad, that it is very unpleasant for me to write, so must request you to inform me of anything that concerns my property, although I do not make the inquiries. I am very sorry we ever heard that Howard had the house. How is it this Mr. Prichard pays the rent for him? Inform me in your next letter. I must confess I should not like to take less rent for it; certainly it is worth the rent I have for it. How do the new houses look? What sort of gardens have they? I shall feel obliged by your informing me that. My letter was opened, and resealed with yellow wax. I did it myself, having forgot to put all in my letter. I intended to keep a strict look out after that fellow William Morgan, and endeavour to learn if he keeps up the insurance on the house, as I wish particularly to know. Howard is liable to pay rent for the house, having taken it in May. Your immediate answer will much oblige, Yours, in haste,

"Catherine Cumming."

"Please direct, as the last letter, to Mrs. Hunt, General Post Office, London."

According to the correspondence I have had with her, she appeared to me to be of sound mind. I appeared as a witness on behalf of the petitioners the last time.

Cross-examined.—I did not on the last occasion express an opinion that I considered her of sound mind. My father was her agent before me. I was dismissed by Mrs. Cumming in March 1844. I received a letter from Mr. Dangerfield. I do not recollect hearing Mrs. Cumming say at the last commission that she dismissed me because I was soft. I went over the property in 1844 with Mr. Dangerfield's brother: a portion of it was almost in ruins.

Re-examined.—The following extract from a letter is taken from the letter received from Mr. Dangerfield referred to in cross-examination:—

"68, Chancery Lane, 14th March, 1844.

"I beg to acknowledge, &c. You are aware, perhaps, that Mrs. Cumming is on many, perhaps on most, occasions in the habit of acting upon her own views and judgment; and certainly as regards the continuance of yourself as her agent, she has not asked any advice from me, &c. &c. J. Dangerfield."

Upon the last commission, I was not called upon to speak to Mrs. Cumming's state of mind at all. Some of the houses I have spoken of are very old. If I had only a life interest, I would not lay out any money upon such house property. I believe the Stow Hill house was uninhabited for some time, in consequence of a notice that the Water Works Company would have to take it.

By the Commissioner.—The income would have been increased from 500*l.* to 550*l.*, if the property had been put in repair.

By a Juryman.—It would have required 1000*l.* to be laid out to produce the 50*l.*

A Juryman.—Then it was not worth her while.

*Joseph Charles Evans*, examined—My wife was a servant to Mrs. Cumming, in 1844, for rather better than three months. I stayed at Mrs. Cumming's house at night. She always acted in a rational kind of manner. The house was in a perfectly cleanly state. I have heard Captain Cumming use bad language towards my wife. My wife left on account of the captain. I insisted on her leaving.

Cross-examined.—The witness was cross-examined about his occupation, and his opportunities of observing Mrs. Cumming. His statements were the same as in his examination in chief.

*Anne Evans*, called.—(It having appeared that this witness had been present in court during the delivery of a portion of Mr. James's speech—her evidence was objected to by Sir F. Thesiger, and was excluded.)

*John Thomas Stocken*, examined.—A hairdresser: knew Captain and Mrs. Cumming, from the beginning of October to the end of December, 1845. I was in the habit of shaving Captain Cumming three or four times a week. There was every appearance of cleanliness in the house. There were two beds, two easy chairs, and three or four others. Mrs. Cumming's conduct was rational, for anything that I saw. I have heard Captain Cumming swear. I saw him supplied with provisions and brandy and water.

*Matilda Cramer*, examined.—In 1845 and 1846, I was forewoman to Mr. Pearson, a confectioner. Captain and Mrs. Cumming was in the habit of coming to the shop. They came in their carriage. I served them with refreshment on many occasions. Captain Cumming was usually served with mock-turtle, gravy-soup, and jellies. Mock-turtle was sometimes sent home. The conduct of Mrs. Cumming towards the captain was nothing different from that of a lady towards her husband. She conversed rationally. I should not have taken her to be insane. Mrs. Cumming paid me the bills regularly. I saw her pay an account to Mr. Pearson about April 1846. I saw nothing different in her conduct. She called upon me after the last commission, to thank me for my attendance at the Horns tavern.

*Elizabeth Buck*, examined.—I am fourteen years of age. My father and mother are both dead. They lived in the service of Mrs. Cumming, in Belgrave Terrace. The captain was rather violent sometimes. He once got up a stick to strike my mother, and missed her and split the table. I remember the police being called, because he wanted to take the keys from Mrs. Cumming: he was very violent before the police came. Mrs. Cumming kept four cats: they were let out every day. Mrs. Cumming's room was cleaned thoroughly once a week, and swept every morning. I remember Mrs. Cumming being taken to the lunatic asylum: the day before the captain left the house, with the nurse in a cab. Mrs. Cumming was very much troubled about it. In the afternoon, a woman came to the door and rung the bell. She forced her way in, then another woman, and two policemen came: when Mrs. Cumming came, they put a strait waistcoat on her. She cried out "Murder:" they forced her into a carriage, and she was taken away. After she was gone, two policemen and Mr. Ince came: my mother refused to open the door: the policemen forced their way in at the parlour window, and let Mr. Ince in at the door. They went all over the house. At the time Mrs. Cumming was seized, she was teaching me to write in a copy-book. She was in the habit of teaching me: she always told me to be kind to my mother. When Mrs. Cumming came out of the asylum, my mother was again employed by her. Mrs. Cumming always behaved like a lady.

*John Green*, examined.—I was a policeman in 1846. I recollect being sent for to the station-house to go to Mrs. Cumming's about March or April. When I got there, Mrs. Cumming said Captain Cumming had turned her and the servant out of the parlour, and had secured the door. Mrs. Cumming was crying; she was in a very nervous state. I took my staff; she said, "For God's sake do not use that." I got in; the captain made an oath and said, if I came any farther he would strike me with a poker. He had a poker in his hand; it was hot. I should say I should have received a violent blow had I not defended myself with my staff. I got away, finding myself in danger. When I got into the room again he had a knife in his hand. He ran round the table after me, and struck me in the hand. I had a tussle with him, and took the knife from him. He was very strong then during the time the passion was on him.

Cross-examined.—The captain had piled the furniture up against the door. I do not know whether it was to prevent any one breaking in, but it was to keep Mrs. Cumming and the servant out; he had turned them both out. He had been using the poker about Mrs. Cumming, by all accounts. She said that he had. I found it was hot by his hitting me with it.

By the Commissioner.—You had a knock on the head, and a cut on the hand; did you take any steps against him? No, the police authorities do not allow us to do that. I was on the sick-list for four days. He was considered as a madman. I believe the police had had interference since then and before.

Mrs. Cumming was very rational, for all I could see of her. She knew perfectly well what she was saying and what she was doing.

*John James Martin*, examined.—I was in Mrs. Cumming's employ about Christmas 1846. I was with her about ten months. She used to give me the orders for the various tradesmen who called. If I paid any bills I put it down in a memorandum, and gave it to Mrs. Cumming at the end of the week, and she looked it over, and if she found it right she would pay me; if she disputed anything she would inquire about it. I always found her behave as a rational person.

Her conduct was that of a lady. There were three cats. The house was not in an offensive state in consequence. The whole house was open to them.

Cross-examined.—Mrs. Cumming was visited in Camberwell-road by Miss Hunt, Mrs. Cook, Mrs. Hutchinson, and Mr. Haynes. While I have been waiting at dinner, she has talked to me about her daughters. She has told me that they wanted to take her away again; and, likewise, if I was to see them come I was not to let them in without her order; she was confident they wanted to poison her to get her property. When she was dining in the dining-room, she would have a cloth laid on the floor, and a plate for the cats, and a clean knife and fork to cut the meat up for their dinner.

Re-examined.—She said, if they came to the house I was not to let them in; that all they wanted was her property, and did not mind poisoning her to get it.

By the Jury.—Was there a knife and fork for each cat? No, only one to cut the meat.

When she talked about her daughters poisoning her, did she appear flighty? She appeared afraid that they were always coming to the house.—Did you think her manner was that of a mad-woman! No, I saw nothing of madness or wrong about her, in any one shape in the world. Her dinner was cooked in the kitchen.

*John Green*, examined.—I am a coachman, in the employ of Mr. Richards, of Camberwell; he is a job-master. I first knew Mrs. Cumming, I think, in August 1846. She was then staying with Mrs. Hutchinson, of Vauxhall. I used to drive her. I was in her employ a year and three months. She presented to me the appearance of a lady of perfectly rational mind. She was very particular about the carriage and horses. Her directions were those of a sensible lady-like person.

A conversation here ensued about the propriety of the jury again seeing Mrs. Cumming. Dr. Caldwell intimated that she was sitting up and expecting the jury. He hinted that the last interview with the jury was too long. The foreman said, the length of the interview, no doubt, was distressing. It was ultimately arranged not to visit her on this occasion.

Cross-examined.—I used to drive Mrs. Cumming to Mr. Haynes's, to Camberwell to Mrs. Cook's, to Mrs. Hutchinson's, and Miss Hunt's, and Mr. Farrer's. She was sometimes late out. She never talked to me about her daughters.

By the Commissioner.—At the Queen's-road she always paid me weekly—every Saturday night, a guinea. I did not board in the house.

*Mr. Joseph Haynes*, examined.—I am a solicitor, in the firm of Carlon and Haynes, St. James's-street. I am the brother of Mr. Robert Haynes. I was first introduced to Mrs. Cumming in November 1846. She told me that she had been induced to make an arrangement on the execution of the Commission of Lunacy in September, three months before, which she had been advised she ought not to perform. She gave me instructions; I took proceedings in consequence of that interview. She said she had been advised by counsel. She said she had been induced to make that arrangement, believing that she had a lesser interest in the estate than it afterwards turned out she had. The deeds were in the possession of Mr. Hooper, of the firm of Saxon and Hooper, mentioned in the arrangement of 1846. She was emphatic upon the subject of not carrying out the arrangement. She gave me a written authority to resist all measures that might be taken to enforce it. She related to me certain facts as to the issuing of the Commission, as to her having been taken away, as to the house having been ransacked and the deeds taken away. I had an interview with Mr. Turnley, the solicitor of Mr. Ince and Mr. Hooper. He requested that I would use my influence with Mrs. Cumming, to induce her to carry out that arrangement, subject to any modification I might suggest. In reference to that suggestion for a compromise, I wrote to Mrs. Cumming. I saw Mrs. Cumming at my office on the 19th of January, 1847. She refused to have anything to do with the agreement. By an arrangement with Mr. Hooper (Saxon and Hooper), an action of *detenu* was brought for the deeds. By an interlocutory order the deeds were deposited with a Master of the Court, and Mr. and Mrs. Hooper were made defendants, and Mrs. Cumming the plaintiff, to try the right to the deeds. My partner and myself wished to see Mrs. Cumming's examination by the Commissioner, in order to make up our own minds. She promised to send it. She called on the 25th of January, and brought it with her. I made certain observations upon it, and she gave explanations. We were satisfied, and went on with the business. On the 3rd of February, Mr. Turnley called at our office and made something like a specific proposal for a compromise. We communicated that to Mrs. Cumming. Mr. Ince and Mr. Hooper were to withdraw all opposition to her recovering the title deeds, &c., provided she would enter into a deed to give her grandchildren one-half of the property at her decease. She called upon us on the 5th; I could not induce her to enter into the proposal. She stated that her family had so behaved, that she would not be coerced into any arrangement.

(A discussion of a somewhat acrimonious character here arose between counsel, from Sir F. Thesiger's objecting to the witness's relating the history of the proceedings in which Mrs. Cumming had been engaged.)

A rule was obtained (on affidavits by the defendants) to stay proceedings in that action. This was communicated to Mrs. Cumming. She made an affidavit in consequence. Her description and her residence were given in that affidavit. The rule was dismissed with costs. Mrs. Cumming was ready in the neighbourhood of the court to be seen by the Judges, and by the Attorney-General, who was her counsel. She was not produced because Mr. Watson, who obtained the rule, gave it up—the affidavits were completely answered. The issue came on for trial in May, 1847. The result was a verdict for Mrs. Cumming. A motion was made for a new trial; a rule nisi obtained. That was discharged with costs. On the 4th Oct., 1847, we received a letter from Mr. Turnley, containing a proposal for a compromise. I sent a copy of that letter to Mrs. Cumming. On the 13th Oct., eight days after the letter was sent, I received an answer from Mrs. Cumming, the whole of which is in her handwriting:—"Gentlemen, Having received your letter enclosing one from Mr. Turnley, I beg again to state that I have long since resolved, in consequence of the wicked and cruel persecution I have suffered from my relations, to consent to no arrangement whatever." At that time the rule for a new trial was pending. We got possession of the deeds. In May, 1847, Mrs. Cumming was served with a subpœna in a chancery suit by Mr. Ince. No bill was filed. The subpœna ran out. On the 6th July, 1848, Mr. Hooper filed a bill. (The prayer of it was to call upon the defendant, Catherine Cumming, to elect under the terms of a settlement by her late father, either to confirm or to renounce the title to certain property). A negotiation was entered into with Mr. Turnley, the consequence of which was, that a decree was made by consent. We have prepared a draft will for Mrs. Cumming. During the proceedings (above referred to), Mrs. Cumming frequently mentioned to me the subject of her will. She stated to me the amount of certain legacies. I was determined not to prepare the will without receiving further instructions. On the 7th June, 1847, we received a letter signed by Mrs. Cumming, but in my brother's handwriting, containing directions. I sent that letter as instructions to counsel to prepare the draft. The draft was sent to Mrs. Cumming. The will was frequently the subject of conversation with Mrs. Cumming. She called at our office alone. I said the will had been prepared by counsel in accordance with instructions sent by her, but that I felt reluctant to prepare the will because my brother was a legatee, and that I should like her further to consider whether she would not make some alteration. I particularly recollect telling her that I thought her grandchildren ought to have something left to them. She said I will not leave the members of my family anything; they have not behaved as children to me, and I will not leave my property to them. I again told her that I thought she ought, notwithstanding what had taken place, to leave something to her grandchildren; when she said, "Mr. Haynes, you are my solicitor, but don't be my dictator." I told her I would obey her instructions, but determined at the moment that I would not. I said, when you are no longer here, a great deal will be said by your giving so much money to my brother; she said she owed my brother a deep debt of gratitude, for that if it had not been for him she would then, if she had been alive, have been in a lunatic asylum. On the 21st July, 1848, she sent for me. She gave me instructions to make certain alterations in the will. I returned the draft to her with the alterations she mentioned on the 4th August. Nothing further was ever done about that will. The red ink alterations in the draft now produced are not in my brother's handwriting, but by one of our articled clerks in our office. To my knowledge that will has never been executed.

The witness then proved the sale to Mrs. Cumming of the two houses in St.

John's Wood. The mortgage on them was 975*l.*, the ground-rent 10*l.* on each house, the purchase-money was 625*l.*, making 1600*l.* The witness prepared the conveyance from Mr. Robert Haynes to Mrs. Cumming on those terms in the ordinary form.

Cross-examined by Sir Frederick Thesiger.—The witness was questioned as to who was the counsel consulted respecting the validity of the arrangement of 1846, and as to whether he was not a personal friend of Mr. Robert Haynes. The witness replied that Mr. Southgate was consulted, but he was not aware that he was on visiting terms with Mr. Haynes. Mr. Southgate stated, that he had never been inside Mr. Haynes' house since he was born. Sir F. Thesiger withdrew what he had stated; he had been wrongly instructed.

My brother assisted me in Mrs. Cumming's business after we became concerned for her. Three sales of portions of Mrs. Cumming's property were conducted by Carlon and Haynes—viz. the sale to the South Wales Railway Company (2000*l.*); to the Newport Water Works Company (750*l.*); and the sale to Mr. Gething, (850*l.*) (Of the 2000*l.* the proceed of the sale to the South Wales Railway Company, 560*l.* had been paid into court). We ceased to be her solicitors before that was payable. The 850*l.* from the sale to Mr. Gething was received by Mrs. Cumming herself in my office; she came in her carriage; no one was with her. Our bill of costs to Mrs. Cumming is 475*l.* There is a balance due to us of 67*l.*

The further cross-examination was upon the subject of the will, and the sale of the houses in Queen's-road. It simply elicited a repetition of what the witness stated in his examination-in-chief.

Re-examined.—The Attorney-General, the present Chief Justice of the Common Pleas, when attorney-general, and Mr. Lush, were consulted as to the validity of the compromise, and gave a very strong opinion. — Why had you made up your mind that you would not allow the will to be executed except in the presence of her medical adviser? Because she had been the subject of a former commission; it was not in consequence of any misgiving of my own.

Mr. *John Carlon*, examined.—Of the firm of Carlon and Haynes.—On every occasion upon which I saw Mrs. Cumming, she conducted herself as a sane person.

*Francis Francis*, examined.—Entered Mrs. Cumming's service as coachman in February, 1848 ; remained till the end of November. I drove her most days of that time. She was particular, like all other ladies and gentlemen, about the horses, and about the harness being kept clean. I never observed anything in her conduct to induce me to believe she was of unsound mind. I was frequently in the house ; it was kept clean and respectable. There were four cats; they were allowed to run over the house and garden. I travelled with Mrs. Cumming to St. Leonard's. She went by the road in her own carriage. Miss Hutchinson, young Mr. Hutchinson, and a servant-maid went with her.

Cross-examined.—Mr. Haynes came to see her at St. Leonard's, and at Brighton.

Mr. *Simeon Thorne*, recalled.—Had known Sir Matthew Wyatt previously to the transaction in which he was concerned for Mrs. Cumming.

*William Gaywood*, examined.—Was a coachman in Mrs. Cumming's employ from October 1848 till May 1850. Mrs. Cumming engaged me herself. I drove her out about three or four days a week. I went into Wales with her in October, 1849. She went in her own carriage all the way. Mrs. Cumming paid all the expenses on the way. She was always very correct, indeed, upon the bills. She has complained at small houses, that they charged her more than at the large hotels. She stayed some time at Newport. Then she went to Bassaleg. She was in the habit of driving out and visiting in the neighbourhood. She was there about six months. She visited her estates. She walked about and saw the farm-buildings. She returned to London by the road, never by the railroad. I have heard her say she disliked the rail. While at Bassaleg I posted a letter from Mrs. Cumming to Mr. Haynes concerning some land the Rhymney Company had got possession of; Mr. Haynes came down. I never observed any indication of Mrs. Cumming's not being perfectly rational and sensible in every respect. I never saw the slightest symptom of insanity.

Cross-examined.—I have been out (driving her) as late as seven or eight o'clock. I am quite sure I have not been driving her about as late as ten or eleven, unless she has been dining out. I have heard her say her daughters behaved very bad to her; and if they ever came there, I was to be sure not to let them in, for she would not see them. She had told me they had ill-used her, and put her into a madhouse, and she was afraid they might do it again. She never mentioned anything about poisoning to me. She has told me not to admit Ebenezer Jones.

Re-examined.—I had told Mrs. Cumming I had met Ebenezer Jones in Newport, and he wanted me to bring one of her daughters into the room with her. He told me if three, or four, or five, pound notes might be of any consequence, if I could be the means of introducing them into Mrs. Cumming's presence, I might have it.

Examined by the Jury.—There have been people call at the house who would not give their names, and said they wanted to see her, and she has told me not to admit them. She said they might be her daughters, and she would not see them unless they would send in their proper names.

*Elizabeth Clarke*, examined.—I went into Mrs. Cumming's service in February, 1849, and stayed till April, 1850. I went with her into Wales. I was present when her tenants came to her. Mrs. Cumming received her rents from them. She counted the moneys and gave the receipts. She visited various people she had known in her childhood. I was with Mrs. Cumming constantly during the whole time. I never discovered anything to lead me to suppose she was of unsound mind. She was particular in money matters. The house was clean. The cats were not at all confined to the room. She has talked to me of her daughters. She said she should have been a kind and dutiful mother to them, if they had been kind to her. She frequently spoke of their having shut her up in a madhouse. She has given me orders never to admit Mrs. Ince or Mrs. Hooper if they came. I recollect a lady calling one day; she asked to see Mrs. Cumming; she came in the name of May. I gave a description of her to Mrs. Cumming. She believed it to be Mrs. Ince. She said she did not know such a name, and not to admit her. I saw Mrs. Ince once. I believe it to be Mrs. Ince. In the beginning of February, 1851, I was sent for by Mr. Haynes to go and attend upon Mrs. Cumming. She said that she had been very ill, and that she had been very ill-treated by Mary Rainey. That Mary Rainey had tied her down with a shawl. When I went, the house was not so clean as usual; there was some cat's dirt about. Mrs. Cumming complained of it, and begged me to clean it. I remember Mr. Thorne calling. I told Mrs. Cumming. She told me to say she was very ill, and could not see Mr. Thorn. A few days after, she sent for Mrs. Hutchinson. She begged Mrs. Hutchinson to let her go to her house, for protection from her children. She expected they were going to take her again to a madhouse. Several persons called about this time; some would not leave their names. I remember while at Mr. Hutchinson's Mr. Ebenezer Jones coming with the police; it was after two o'clock. At that time Mrs. Cumming had been confined to her bed for five days. He came to the door of Mrs. Cumming's room. Upon my refusing to admit him he burst open the door; he gave her in charge of the police. A medical man represented to the police that it would be dangerous to remove Mrs. Cumming.

By a Juryman.—She said there were people slept in the house (Herbert Villa) which I heard myself on the Sunday night. I saw a bricklayer go out about six o'clock. I believe it was a man of the name of Hickey.

By the Commissioner.—Her meat was all cooked in the kitchen. Witness believed that the man she saw go out of the house on the Monday morning was Hickey; and that it was the same man who called again in the evening for a hammer; witness's husband gave him the hammer.

*George Clarke*, examined, the husband of last witness.—Went into Mrs. Cumming's service about March, 1849, left in April, 1850. While Mrs. Cumming was absent in Wales I had charge of her house in the Queen's-road. While she was at home she gave the orders; she sometimes gave me money to lay out for her. She was particular in demanding an account. The cats were left behind when Mrs. Cumming went into Wales. I received orders not to admit Mrs. Ince and Mrs. Hooper, Mr. Hooper, and Mr. Ince, and Ebenezer Jones. She described Jones to me. I left of my own accord, as I was taking a greengrocer's shop. From May 1850 to May, 1851, my wife and I occasionally called upon Mrs. Cumming. I remember in February, 1851, being requested by Mrs. Cumming to stay at Herbert Villa. She gave as a reason that an Irishwoman (Rainey) tied her down in a chair. On the Sunday night I heard some men talking below. When I was searching below, Rainey told me I had no business in the kitchen, concerning myself about her business. I told Mrs. Cumming there was some person in the house; she told me to look; I went down, but could find no one. The following day Mary Rainey came back again for her wages. Mrs. Cumming said she would pay her if she gave a receipt. Rainey said she could not write. Mrs. Cumming then said she was to go to Mr. Haynes, who would pay her. She came again the next day for her boxes and wages, and said she would go up stairs and be paid. Mrs. Cumming told me to go down and prevent her. (Witness repeated some foul language that Rainey used to him.) She said, "Last night I diddled you, for I had two policemen in, and another man, all the night." Shortly after that a man came and gave some name, and said he was the landlord of the house. I told Mrs. Cumming the name. She said that was not the name, for Sir Matthew Wyatt was the name. She said I was not to admit him. Then he said he would get over the wall. I said if he did I would knock him down. He joined another man who had on a hat like the one described (by Mrs. Cumming as worn by Ebenezer Jones). I have seen Jones up here. I cannot swear it was him. There were three cabs below. I saw them join some ladies; they got into those cabs, and went away together. On the following day I received some information from Mrs. Cumming's butcher. Mary Rainey had told him how her daughters were coming after her to take her to the madhouse. I told this to Mrs. Cumming. On the following day Mrs. Cumming went to Mrs. Hutchinson's. Mr. Hutchinson came on the following day and I helped to remove the furniture. During all the time that I was at Mrs. Cumming's I never saw anything to induce me to believe she was of unsound mind, quite the reverse.

Cross-examined.—(The witness was questioned as to his occupations since he left Mrs. Cumming's service; as to whether he had killed one of the cats, and as to the removal of the furniture.)

The *Rev. Hugh Williams*, examined; the Rector of Bassaleg, a magistrate of the county of Monmouth, and the Chancellor of the Diocese.—As Chancellor of the diocese I have to adjudicate upon testamentary causes; and I have been fortunate enough never to have my judgments reversed, and I have presided in Llandaff Court for thirty years. My duties involve the necessity of deciding on the mental capacities of testators. I have known Mrs. Cumming since September 1849, when I became acquainted with her personally. I have known her as my landlady, by correspondence with her agents, previous to that. She came to reside in my parish in September, 1849. She attended Divine service in my church on the 14th of October. Her demeanour and conduct while in church were perfectly proper; she sat nearly opposite to me, and I had every opportunity of observing. I had heard at that time that a Commission of Lunacy had sat to make inquiry with regard to her sanity; I was consequently prepared to watch anything that might occur indicating anything like aberration of mind. I invited her to stay at my house one evening; she spent the evening there on the 20th of September, 1849. She joined in the ordinary conversation; my wife and family were present. I saw her also on the 10th of September. I called upon her to know to whom I should pay my rent. Her answer was, through the servant, "To myself; I will call to-morrow at your house." On the morrow I met her in the village, in her carriage, coming towards my house; I went up to her; I paid her there. I occupied under her a small field. I had stated to her that I thought my rent was too high by a great deal; she very quickly found arguments against reducing the rent, and said, finally, that she would refer me to Mr. Haynes, her agent. Throughout my communications with Mrs. Cumming, I certainly did not observe the slightest symptom of insanity, and I watched her closely.

Examined by the Jury.—I got my rent reduced afterwards, but not by Mrs. Cumming; it was by Sir Charles Morgan; it is part of the property Sir Charles Morgan bought.

*Lewis Edmonds, Esq.*, examined.—I am now upwards of 70 years of age. I have been twice mayor of Newport; I am still an alderman of the borough. I have known Mrs. Cumming for forty years; I remember her coming down to Newport in September, 1849. I remember calling on Mrs. Cumming, at Bassaleg, to ask her permission for my grandson to sport over her estates. I had a long conversation with her; I was aware at the time that a Commission of Lunacy had been issued against her. I discovered no symptom of unsoundness of mind; she appeared to me a very shrewd woman indeed. I told her that a preacher from Newport would preach at Bassaleg on the following Sunday; she said, she would certainly go to the chapel. I noticed her demeanour at chapel; it was very correct and very attentive. She said it was a very good sermon, only that he spoke too loud, which I think myself he did. She requested me to look out for a furnished house for her; she should be very glad if she could get a house in Newport. I saw her, I should think, seven or eight times. On no occasion, whilst I saw her the last time did I discover anything irrational about her, or approaching to it. She said something to me about her intention to sell a portion of her property; she said, "Mr. Edmonds, I have some estates to sell, and you had better buy; I do it in consequence of my daughters treating me exceedingly ill." I have known her forty years. She was always rather of a violent temper.

Cross-examined.—I have not seen anything violent. I have seen her and the Captain cross with one another. She was considered a bad temper. When I first recollect her, she was living with her husband at the Red House. She was very handsome, fond of dress, of very lady-like manners. I saw her about fifteen years ago. I observed no difference in her at that time, only she was growing older. I never talked to her about her family.

Re-examined.—The last time I saw her, she was the same; neat and elegant in her dress, and with the manners of a lady. I saw not the least change in her mind.

(The jury expressed a desire to see Mrs. Cumming. Dr. Conolly intimated that she was very ill.)

*Thomas Evans* examined.—I reside at Bassaleg; I am a dissenting minister. I act as a house-agent; have done so for many years. I became acquainted with Mrs. Cumming on the 29th of September, 1849. I called upon her to collect some manorial rents; she asked me the amount, and paid it. I went with her to see one of her tenants. She asked for his rent; he brought the money out. She said, what is the income-tax, and took the pencil, and made it up, to deduct out of the rent; she gave him a receipt. George (the tenant) said his rent was too high; she asked about the produce, and the price of the cattle, and so on. She asked him if he had not had some repairs lately? he said, "Yes, Ma'am; but not enough." She went with me to look at the buildings. She then went to see my house (which she had proposed to hire of witness.) She went over every room; she took the house; she remained there five months. I showed her the tithe-commutation map. She pointed out to me her farms in each map. She then turned to the referring-book that was belonging to the map, and looked what was the tithe on every field, and then she added up what it was in the whole; and then she said: "Bother the Church, there is plenty of taxes on land, without paying tithes, and the Church ought to support itself." She told me her father was a Dissenter. She showed me some woodland on the map, and wanted me to try and offer it to some gentlemen in the neighbourhood, because they were felling timber there without her knowing it.

(The Commissioner, accompanied by the Jury, Mr. Petersdorff, Mr. James, and two short-hand writers, proceeded to visit Mrs. Cumming—the *second* examination.)

*Thomas Evans'* examination resumed.—Mrs. Cumming authorized me to sell some timber, and I was to deduct my rent out of it; my bill would state exactly what it was. I was with her going over the estate every other day since she was there, unless it was a very wet day; she did behave like a lady, and I never saw anything out of the way. I heard she was in the madhouse, and I was particular in taking notice whether I could find anything wrong in her mind or not. (Witness related a conversation with Mrs. Cumming, in which she related the circumstances attending her confinement in the asylum.) She told me, that although she could not prove it, she had reason to believe one of her daughters had tried to poison her. She told me, she took the milk and it was magnesia that was in it, and that she offered it to the cats, and the cats refused to take it; and that a chicken, in the kitchen drank it, and died. She went several times to my chapel and heard the children sing. She gave me five shillings to buy small books to reward them. I saw her write a letter to Mr. Robert Haynes on the 27th of November, asking him to come down about some property. One day, in my house she did ask me, (looking through the window, and seeing people working on the other side of the river) "cannot I see some people working there?" I said, "yes, Ma'am." "Is not that my land? What business have they got to do with that land?" I told her, I thought that Ebenezer Jones sold an acre and four perches to Mr. Crawshay Bailey, and she said that she had never authorized him to do such a thing. She said to me, "who is the agent of Mr. Bailey?" and I said, Mr. Brewer; and she told me, then will you go to Mr. Brewer, and ask him, who authorized him to touch my land? I went to Mr. Brewer, and told Mrs. Cumming what he said. Mr. Brewer told me he gave Ebenezer Jones 6*l.* to come up to London, to buy this property if he could; and that he sent three letters from London that all was ready; that Mrs. Cumming was willing to sell the land for 50*l.*, and gave him 5*l.* for selling it, and for them to begin when they liked to cut the land. She denied this, and wrote to Mr. Haynes. (Mr. James read the letter:—

"Dear Sir,—I should thank you to be at Newport as soon as possible, as they are taking away the land on every side. Surely you can find an opportunity to come and see what is the state, &c. If you will inform me the time, or more properly the day, I will be there to meet you.")

She wrote the whole of the letter herself.

(The witness was then asked several questions by the Commissioner and the Jury concerning the value of the property.)

At the opening of the proceedings on this the 10th day, Sir F. Thesiger observed, that there was a subject to which he ought to call the attention of the Commissioner. It was agreed, he said, between my learned friends and myself, at a very early period of the inquiry, that no questions should be put to the medical gentlemen of this description—"Having heard the whole of the evidence, is it your opinion upon that evidence that —— is or is not of unsound mind?" The consequence was this, that their daily attendance here becomes quite unnecessary, because all they would have to do would be to come and tell us what interviews they had with Mrs. Cumming, and what is their opinion as the result of those interviews. Now, we know that their time is extremely valuable, but we know, also, that the expense of their daily attendance must be very considerable.

Mr. Serjeant Wilkins.—Does my learned friend use it as a matter of legal objection that these gentlemen should not be here? If so, I am prepared to answer it. And when my learned friend talks about expense, he should remember I saw his medical men here day after day, and not a word about expense was urged. If they (the medical gentlemen) have been here, it has been from the uncertainty of the time at which they may be called. But I am sure there is no objection in their being here.

Sir F. Thesiger.—Of course, there can be no legal objection. Sir F. Thesiger then repeated that his objection rested on the score of expense.

A discussion also arose between the Commissioner, Jury, and Counsel, as to the mode and time of visiting Mrs. Cumming. A juryman wanted to know whether they were not at liberty to go to Mrs. Cumming without counsel on either side. Mr. Serjeant Wilkins said, he entreated that counsel should not go. The Commissioner felt that they ought to go; that the lady's evidence was part of the case, both of the plaintiff and the defendant. Mr. Serjeant Wilkins suggested, in order to avoid the semblance of unfairness, that the jury should visit Mrs. Cumming without any notice. In reference to this subject, some observations were also made with respect to the conduct of the servant (Blake), whose appearance in the room (at the time the jury were with Mrs. Cumming) yesterday, the Commissioner said he was bound to take notice of.

*Thomas Evans*, examined.—At the time that Mrs. Cumming took my house, she asked me, "What will you charge for this place?" My answer was, "I do not know, ma'am; I will trust to your honour." Then she said she paid Mrs. Phillips, at Newport, 1*l.* 15*s.* a-week for two rooms. She said she could not prove from whence it (the poison) came; that she sent it to the chemist to see what it was; but that she did believe her own children were wicked enough to put it in. I made no inquiry as to how the children might have done it.

(It was here observed that Dr. Conolly was in the room. Sir F. Thesiger said, I would rather that Dr. Conolly should not be here, on account of the expense of it. It was intimated that Dr. Conolly had no idea of receiving a farthing. Sir F. Thesiger then said there could be no objection to Dr. Conolly's presence, but requested the Commissioner to take a note of it.)

I offered 300*l.* for one lot (of the ground sold afterwards to Sir C. Morgan).

The sale had been advertised in the Welsh papers. Forty or fifty people attended the sale. I wanted the piece for a coal-yard.

*Thomas George*, examined.—(This witness not speaking English, the Rev. Chancellor Williams was sworn as interpreter). Is 75 years of age. Lives at the Blackbird's-nest, a farm belonging to Mrs. Cumming. First saw Mrs. Cumming when she came to his farm in her carriage. (Witness related the details of the interview with Mrs. Cumming described by last witness). He paid Mrs. Cumming his rent. She reckoned the income-tax on the moneys, and said how much remained after she took off the income-tax. Witness paid her that, and got a receipt. (Mr. Evans and witness's wife, who spoke English, aided witness in his intercourse with Mrs. Cumming). She asked him if he could find hay and straw for her horses. He supplied her with provender for the horses. He saw her on other occasions. He could not perceive anything the matter with her; but he perceived that she was more quick and intelligent than himself.

*Esther Blake*, not sworn—examined by the Commissioner.—(Esther Blake is the servant who entered the room when the jury were with Mrs. Cumming). The Commissioner asked her who sent her into the room? She explained that on the previous occasion of the jury's visit, Mrs. Cumming suffered inconvenience, and Mrs. Cumming wished her to come in to ask her to take a little refreshment. She did not come in to interrupt the jury; she should be sorry to treat them with disrespect. The Commissioner said that by so acting she was doing that which was injurious to Mrs. Cumming's case. Esther Blake repeated that she merely went in to ask her to take a little refreshment, thinking she required it.

*Richard Mullock*, examined. — An alderman of Newport; has been mayor. Knew Mrs. Cumming in 1809. She was then Miss Pritchard. Saw her after her marriage. Saw her frequently while she resided at Newport. I remember her at Newport with her daughters when quite young. I saw her again in 1849. She called several times at my house, occasionally on business. She made inquiries as to persons that were old inhabitants of Newport whom she had known. I asked her as to her daughters. She said she believed they were well, but things were not so pleasant between her and her daughters. I was not then aware that there had been any question about Mrs. Cumming's sanity. I observed nothing whatever to lead me to suppose she was of unsound mind. She appeared to possess a rather better recollection than I did of things that had occurred forty years ago.

Cross-examined.—I am a shopkeeper. I did not visit Mr. Pritchard, but went occasionally on business.

Re-examined.—On the last occasion I saw Mrs. Cumming at least half a dozen times. On each of these occasions she conducted herself as she had done on former occasions.

*Miss Mary Hunt*, examined.—Formerly a milliner; now retired from business. I have known Mrs. Cumming many years. I am a bad hand at dates. Cannot exactly say how long. I never knew much of Captain Cumming. I remember, when they were living in Ebury-street, her telling me her husband was in difficulties. She asked me to take in her letters, which I did for some years; they were addressed in my name; it was only the letters she received from Wales. She said Captain Cumming had some gambling friends, and she did not wish them to know their address. They knew his property was in Wales. When she was at Maida-vale she was not friendly with her daughters. I always considered her most lady-like. I never saw her intoxicated. I thought her most particular in her house. I was in the habit of seeing her up to the time of the last inquisition in 1846. Up to that time I had never seen anything to induce me to think her of unsound mind. She was always exact in her accounts. When the inquiry at the Horns Tavern was over, I accompanied her to Mrs. Hutchinson's. (Witness then referred to many other occasions of her seeing Mrs. Cumming since 1846.) Mr. Grove, of Bond-street, is my brother. I am living with him. I have seen Mrs. Cumming since this inquiry began. Mrs. Cumming sent her servant to say she wished to see me in Stamford-street; that was somewhere about February last. She said Ebenezer Jones had broken into her room, and had frightened her very much. I think she said he came to take her to prison for perjury; he took some policemen. She said it was through their (Mrs. Ince's) connexion. She said she did not intend staying there longer than she could get somewhere else to be secure. She thought Mr. Ince's people were watching her. On one occasion she told me

Mrs. Ince had been to see her while she was at Mrs. Oldfield's. She said she had received her quietly, because she (Mrs. Cumming) wished to deceive her. She did not wish Mrs. Ince to know she was in a passion with her, or anything of that sort. She asked me if I knew of any place in the country where she could go to get out of her way; she thought Mrs. Ince was plotting against her. She had an idea that this would occur which has happened. I have known Mrs. Cumming full 25 years. I did not hear that Mrs. Cumming had made a will in my favour till it was mentioned in court (Chancery) two months ago. Before that time I had not the slightest notion that Mrs. Cumming had left me a farthing. I gave my evidence at the last inquiry at the Horns Tavern (1846).

Cross-examined.—I am prepared positively to state that I did not, upon the last inquiry, say that I had not seen Mrs. Cumming for six or seven years. I have always found her exactly the same during the whole course of those years (25 or 28). I do not know anybody whose conduct throughout has changed so little. She was always neat in her person. Her house always clean. She was always fond of cats. When she was at Maida-vale she spoke of her daughter, Mrs. Hooper, who was always rather a favourite of mine, and I wished to make them friends; and Mrs. Hooper was confined, I think, with her second child; and I always said what I could to make them friendly. I thought they were a very united family till the marriage. She was very much annoyed about Mr. Hooper's marriage, and never liked Mr. Ince. She appeared very much to regret that differences had arisen between her and her daughters. I saw her several times in Belgrave-place. She was the same—lady-like, clean. I think I did not go into her bed-room (there). I went with Mrs. Hutchinson and Mrs. Cumming from the Horns after the Commission. I had not known Mrs. Hutchinson before.

By the Commissioner.—When she got out of the carriage, and was in Mrs. Hutchinson's room (after coming from the Horns), she was vexed about having come to some arrangement, that she had agreed to the papers, or something doing away with the trial, because she thought she might not have been brought in insane. — Can you remember any other circumstances that you think might lead her to dislike her daughters? Mrs. Cumming has told me she has been vexed about one thing or another. Once she was very vexed about that plate and silver that Mrs. Ince was to have bought for her. I understood Mrs. Cumming she gave Mrs. Ince some money to buy, I think, some family plate for her, and she was vexed she did not get the things she expected to have. — I saw her about ten days ago. She remembered things a good deal better than I did. She was very much worse in health than when I saw her before. She complained. She said it was all Mrs. Ince and Mr. Jones's doing that she was dragged about so. When she was at the Horns Tavern she complained of Mr. and Mrs. Ince and Mr. Hooper, not of Mrs. Hooper. — Have you ever heard her speak of her grandchildren? No. I have tried to make her friends with them because of her grandchildren; and Mrs. Hooper's little girl is so like her grandmamma that I was in hopes they would have been friends. I think, if she had been left quite alone after she came from the Horns Tavern, in course of time she might have been brought round by friendship, and that.

A Juryman.—How do you mean, left alone? I mean if Mr. Ince's people had not annoyed her.

*Eliza Rosina Cooke*, examined.—The wife of Mr. Cooke, wine-merchant, of Cannon-street. I became acquainted with Mrs. Cumming in 1846 at Mrs. Hutchinson's, whom I had known for some years previous. I visited her on many occasions. She always behaved in a most ladylike, rational way. I have seen her preside in her own house. I have seen her make purchases. She made them as a shrewd, intelligent woman. Her house has always been in good order whenever I have visited her. I have allowed my daughter to stay there for ten days. I think she is perfectly rational and of sound mind.

Cross-examined.—She never spoke to me about a will. She never said anything to me about the poison.

By the Commissioner.—She was always quite calm when she spoke about her daughters. I never saw her excited.

*Leopold Fischel*, examined: A commission agent, living in Fenchurch-street, the son-in-law of the last witness.—I became acquainted with Mrs. Cumming in January, 1848. I visited her several times. She presided at the head of the table as a lady generally does. She is as sane, I should think, as I am.

*James Kell*, examined : A fly-proprietor in St. John's Wood.—I was coachman to Mrs. Cumming from the 19th of April to the 12th October, 1850. There were four cats. I have seen them in every room in the house, and in the garden. I have had money from Mrs. Cumming to pay bills with. I gave the receipts to her. She was particular in seeing they were correct. I never saw her behave in any way irrational.

*Esther Blake*, examined: A nurse in the service of Mrs. Cumming since she came from Effra Hall.—I had been in her employ before, last April twelvemonths. Mrs. Cumming engaged me herself. I was with her three months. At times she could not be otherwise than dirty, her illness was so great. She was very sorry for it. Mrs. Cumming gave all the orders. She never paid any bills without having a receipt at the time. If there was a halfpenny or a penny not quite correct, she would point it out. The cats were not confined to her room. A charwoman came sometimes. The house was cleaned throughout. The carpets were beat. She particularly desired me to let no one into the house without taking their name up into the bed-room. There was a person came to the house. I think she told me her name was Ince. I judged her to be Mrs. Cumming's daughter, because she was rather like her. I described her to Mrs. Cumming. She said: "By no means let my daughters into my house—they will take me to the madhouse, as they did before." When Mrs. Cumming returned from Effra Hall she was very ill indeed. She complained of pain in her limbs, and the treatment she had received. She said she was dragged from her house like a felon. She has mended by degrees since her return, but for some time after she came from the asylum I was very doubtful whether she would ever recover again. She is rather of a bad temper. I could not mention any instance that I thought her in any way insane.

Cross-examined.—Mrs. Moore called almost every day. Mr. Haynes did not come without being sent for. I never heard her say anything about poison.

By Mr. Serjeant WILKINS.—During this last time a great many medical men have visited her. I should think ten or twelve. After their examinations she has appeared very much fatigued.

By the COMMISSIONER.—I gave her a little sherry and water or port wine warmed, sometimes every half hour. I do not think any medical man has stopped more than twenty minutes. I have always been in the room.

*Charles Crane*, examined.—Was coachman to Mrs. Cumming in December, 1850. I remember Mary Rainey. She was not of a mild disposition—very violent. I know that she was acquainted with John Hickey, an Irishman. Hickey's daughters came shortly after. Before this Mary Rainey came into the house, it was very quiet. As soon as she came, there was nothing else but disturbances. The night before removing to Howley Villa, I remember Mrs. Cumming ringing the bell. Me and Mary Rainey both went up. Rainey went up in her clogs. Mrs. Cumming said, she thought it was improper. I had not heard of any scream or cry from Mrs. Cumming before that. I consider Mary Rainey's conduct to Mrs. Cumming otherwise than respectful. I have heard Mrs. Cumming order Mary Rainey and likewise Mrs. Hickey's daughters to clean up the cats' dirt, and they told her they would not. Mary Rainey has offered me money twice to go to Mrs. Ince's. Before Mary Rainey came, the cats were allowed to go all over the house. I have seen her drive them up stairs again. I remember one Friday night, when the police came, I was sitting in the kitchen with Mary Rainey. Mrs. Cumming rang, and Rainey answered it. Shortly after, I heard a scream of murder. I ran up stairs. Mary Rainey was in Mrs. Cumming's room—the table was knocked over. Mary Rainey was in a great passion. Mrs. Cumming told me to request Mary Rainey to know what her wages were, and she gave me the money to pay her, and send her out of the house directly. She said that Mary Rainey had been ill-treating her. She refused to take her money. We came down stairs. Mrs. Cumming rang the bell a third time. She went up stairs. I heard Mrs. Cumming "cry out" again. I met Mary Rainey coming down stairs. I let the policemen in. They went up stairs. They knocked at the door, and Mrs. Cumming, I believe, refused to open. They asked her for what reason. She said, because she had been ill-treated by the female servant. The police got out on the leads, and got through the window. He stumbled into her room. Mary Rainey fetched a small tool to open the door. After the policeman left, Mrs. Cumming requested me to stay in the room, because she was afraid to be left alone with Mary Rainey. During all the time I was with Mrs. Cumming I never saw anything to induce me to suppose she was insane.

Cross-examined.—I took a note from Mrs. Cumming to Mr. Thorne, and Mr. Thorne came. I recollect on Saturday evening being sent for to her bed-room while she wrote a note to Mr. Longman. She told me that that note was to tell Mr. Longman to take the carriage and horses and keep them until she paid him what she owed him. I took the carriage and horses to Mr. Longman's. — Do you mean to say she did not inquire afterwards what you had done with the carriage and horses? Not to me. Mary Rainey told her she had said so. I was not desired on the morning after the occurrence with the policemen to go to Mr. Thorne's. Mary Rainey did not tell me.

This witness underwent a very long cross-examination about his occupation, the wages he had from Mrs. Cumming, whether he had seen Mr. Haynes, &c.

Did you ever go to Mr. Ince and speak about Mrs. Cumming? I went there once. — Did you say upon that occasion that she was as mad as a March hare? I did not. (The witness's attention was then directed to the two policemen who had been called on the other side (Parsons and Richards.) — Did not you say to those policemen that she was as mad as a March hare? No, I did not. I should not like to swear I did not.

Re-examined.—I know those two policemen. I do not know that they are acquaintances of Mrs. Rainey. I will swear I did not say to them that Mrs. Cumming was as mad as a March hare. It was Mary Rainey who took me to Mr. Ince's; Mrs. Hickey went with us. I did not afterwards go to Mr. Jones's, a solicitor in Sloane-street; they wanted me to call, but I never went. (The witness's conversation with Mr. Ince was objected to by Sir F. Thesiger.) I had never seen Mr. Haynes before I went to him, the night when Mrs. Cumming sent me, in consequence of Mary Rainey's violence.

By the Commissioner.—Was in Mr. Lucas's employ before he went to Mrs. Cumming. Never told him he was driving a mad woman.

*Mr. Stephen Hutchinson*, examined: A civil engineer, and proprietor of the Bromley Gas Works.—I have known Mrs. Cumming for twenty years. I have visited her and her husband at their various residences. They have visited me. I remember a party at my house in 1844, at which Mrs. Ince attended. I remember the time Mrs. Cumming was taken to the asylum; a few evenings before that she had taken tea with my family. On that occasion she conducted herself as a rational being. Up to that time I had never discovered anything to induce me to suspect unsoundness of mind. A few evenings after this Mr. Driver, Mr. Ince's assistant, came to my place. I ascertained from him that Mrs. Cumming had been removed to a lunatic asylum. In the summer of 1846, I spent some time on the continent: on my return I was summoned to attend the inquisition. The matter was arranged before I was called upon to give evidence. I was named as one of the trustees under that arrangement, on behalf of Mrs. Cumming, and approved of by Mrs. Ince. I heard the commissioner, upon that occasion, tell Mrs. Cumming that she was a free agent. I took her home in my carriage to Vauxhall. She remained in my house several months. She was very ill at that time, and was constantly attended by one of my servants, by a medical man, and Mrs. Hutchinson. It was in the early part of 1847, I first heard that Mrs. Cumming had made a will. I was informed (by Mr. Haynes) that I was to be one of the trustees under that will. I said I would decline it, as I had more than I could attend to of my own business. In 1847, I had several conversations with Mrs. Cumming. I endeavoured to persuade her to carry out the agreement which had been made at the Horns Tavern. I was present upon an occasion when Mr. Petersdorff, Mr. Southgate, and Mr. Haynes were present with Mrs. Cumming. The parties then present did all they could to persuade Mrs. Cumming to carry out the arrangement. She said she was determined never to carry it out (that was the 2nd November, 1846.) I consider Mrs. Cumming self-willed. I saw Mrs. Cumming in 1847, 1848, 1849, 1850. She has been several times to my house at Bromley. In 1850, I saw her, by her desire, at St. John's Wood. She told me she considered Mr. Haynes was neglecting her business, and therefore she thought she would employ some other gentleman, and wished me to look into her affairs, as she was short of money. I have known Mr. Haynes since the commission in 1846; not before. I went to Mr. Haynes to see what state her affairs were in. I got from him an advance of 100*l.* She came to my house in Stamford-street, in February (1851). She said she was in fear of being again taken to an asylum, and that was the reason she had left the Queen's-road. She said she considered there was a conspiracy going on with her servant, Rainey. She begged that none of her family might be admitted. I saw her next at Worthing, in September. Upon that occasion she had just received a report from the Lunatic Commissioners declining to interfere, having examined some of the medical gentlemen. She was very much pleased that she was free from her family. I did not see her again till I saw her at the London Bridge Railway terminus, in the custody of the keepers of Effra Hall. It appeared to me that more violence was used than was necessary. She screamed. During the twenty years of my acquaintance with Mrs. Cumming, I have never seen or heard anything to induce me to doubt the soundness of her mind.

Cross-examined.—(The witness was questioned as to the reason for his leaving the Vauxhall Gas Works.—Had there been no complaint about the accounts? The witness was engineer, and had no accounts or books to keep. Witness started the Gas Meter Company, which was now being wound up. He projected the Monetary Loan Company. He did not know where Mrs. Cumming's furniture was removed to from Herbert Villa. Mrs. Cumming took a house belonging to witness at Oxford-terrace, Old Kent-road. The furniture was eventually removed there. The rent was £38 a-year. She had it for half a-year. Witness was paid the rent in an account, including other items, by Mr. Haynes: did not know the amount.)

Re-examined.—Witness never received any pecuniary favours from Mrs. Cumming in his life.

(The account was produced. It amounted to 37*l.* 17s. It included 15*l.* for rent of house in Oxford-terrace; the remainder for various sums expended for Mrs. Cumming.)

*Mrs. Sarah Hutchinson*, examined: The wife of the last witness.—I have known Mrs. Cumming upwards of twenty years. (The evidence of this witness was the same as that of the last, down to the date of the commission, 1846.) I went to see Mrs. Cumming in the asylum at York House. I got in by mere accident. I had applied for an order; it was refused. I went to the asylum a second time, and was refused admittance. I was examined at the last commission. Mrs. Cumming accompanied me home, at her request. She made up her mind immediately not to carry out the arrangement. I tried to persuade her to enter into it. I saw Mrs. Cumming at the Queen's-road, when Mary Rainey was there. Mrs. Cumming complained that she did not do her bidding. I myself observed that her conduct was insolent. Mrs. Cumming expressed her suspicions that Mary Rainey was in connexion with her family. She came to my house again at her own request, about 5th February, 1851. She gave as a reason that she thought they would come and take her away to an asylum, as they had done before. A few days after that Ebenezer Jones came to my house with some policemen. Mrs. Cumming was very ill. It was made known to Mr. Jones that she was very ill. Notwithstanding that he burst open the door of the bed-room, and gave her into the custody of the police. Mrs. Hickey came with the police. A few days after Mrs. Ince came. Her name was sent up to Mrs. Cumming. She sent her servant to deny her. Mrs. Ince did not go away; she kept knocking at the door. Mrs. Cumming told me afterwards that the warrant before Mr. a'Beckett was dismissed. This proceeding of Mrs. Ince and Mr. Jones alarmed Mrs. Cumming very much; she wished to change her residence; she went to Mrs. Oldfield's. Mrs. Cumming herself gave the order to remove her furniture (from Herbert Villa.) It is the same furniture that is in her house now. I saw Mrs. Cumming at Mrs. Oldfield's; she stated to me that Mrs. Ince had followed the servant up, and rushed into the room. She ran to her, and put her arms round her neck. She was very much alarmed, and thought she would do her some bodily harm. (Witness conversed with Mrs. Cumming afterwards upon that interview.) She said that from her own observation, she saw Mrs. Ince take notes of everything about the room, which created great alarm in her mind. She did not think she intended to be so friendly as she pretended to be. The following day, or a day or two after, I was at Mrs. Cumming's, and during the time I was there, Mrs. Ince and Mrs. Hooper, and several others, came to the door; they were traversing the street opposite, and speaking to several persons. Mrs. Cumming pointed them out to me herself. They rang.

The message came up while I was there, and Mrs. Cumming desired me to write a note to say she would not. Among the persons I saw with Mrs. Hooper and Mrs. Ince, was a policeman. After this Mrs. Cumming again expressed a desire to get away. She requested me to get from Miss Hunt an address at Southall. She went there for a month. I next saw her at Worthing. She passed there under the name of Cleveland. She said that her daughters were pursuing her, and she wished to change her name on that account. I was present on one occasion, when Mrs. Cumming expressed her delight at being declared a free agent by the Commissioners. She accordingly went to Brighton and resumed her own name. I did not see her at Brighton until the 28th October, when she was again apprehended. When I arrived there I found several persons in the parlour—some police. Mrs. Cumming was in her bed-room: she was very much agitated. She said some gentlemen had been into the room, and had been asking her questions, and she hardly knew what she had said—that they had broken open her door. She was in continual alarm. She expected to be forced away every minute. On the following day (Wednesday) Dr. Hale saw her. On Thursday Dr. King called. He came to the door (of Mrs. Cumming's room) and demanded admittance. Mrs. Cumming told him she had seen her own medical man, and did not want any more advice. Dr. King said if she did not open the door he would bring the police. Immediately after that the door was forced open. Dr. King, the policeman, Mr. Chase, the superintendent, a female keeper, and several others came in. Dr. King ordered her immediately to get ready. He said he had the Lord Chancellor's power to take any body into custody who interfered. He was very rough. I saw Mr. Turner. Mrs. Cumming asked Dr. King where he was going to take her to. He said to an asylum, and she would know when she arrived. When I requested them to tell me, they gave me a wrong address. She said she had never been on the railway before, and was much alarmed. I saw her when they brought her on the platform (at the station.) They dragged her—she had not a foot on the ground; she appeared suffering much. During the whole time I offered no obstacle. I applied at Effra Hall next day to see Mrs. Cumming. I was refused. I saw her on the evening when she left the asylum. About a week before this inquiry, I again saw her by permission of the Lord Chancellor. It was during the time she was at my house, in May, 1847, I first heard about a will. She was very ill; she expected to die. She requested me to write to Mr. Haynes for his attendance. Mr. Haynes had an interview with her. After that interview he told me my husband was to be one of the executors. He did not tell me she had left me or my family anything. My husband refused to act. The first time I heard the contents of the will was not until the last twelve months; that was the time when Mrs. Cumming consulted Thorne.

Cross-examined.—She said she had employed Mr. Thorne because she thought Mr. Haynes was neglecting her business. About the latter end of the year she began to be suspicious about Mr. Thorne, because he would not return her papers. I remember Mr. Thorne coming to my house in January, 1851, while Mrs. Cumming was there. He told me he was her solicitor. I did not refuse him ; Mrs. Cumming refused. I wrote a letter at Mrs. Cumming's request. Mr. Haynes had been with her; but many days before. I was a music-mistress for a short time. I taught the Misses Cumming. No will was ever executed in my parlour. Mrs. Cumming never told me about the execution of a will. She only told me my husband was executor.

By the Commissioner.—I came in the same train, not in the same carriage, with Mrs. Cumming, from Brighton. The parties promised Watson, her servant, should go with her; but they would not let any one go. Mrs. Cumming is very much weaker in bodily health of late. — Q. You tell us you think this lady's memory is not so good as it was? A. Only since she has been at Effra Hall. Before that I had not observed any change in her.

*Mr. Robert Crooke Romsey,* examined.—A solicitor. I acted as the agent for Mr. Turnley, of Cornhill, in the conduct of the prosecution against Mrs. Cumming for perjury. On the 11th of February, 1851, I received a note from Ebenezer Jones, requesting me to attend at Mr. Turnley's office, to receive instructions. I went in with Ebenezer Jones, and there we met Mr. Ince and Mr. Turnley's clerk. We then discussed the mode of carrying on the prosecution against Mrs. Cumming. A written case was put into my hand. (Sir F. Thesiger here interposed. He contended that what took place at the interview in Mr. Turnley's offices was irrelevant. A discussion ensued. Mr. Serjeant WILKINS urged that Sir F. THESIGER in opening his address to the jury, endeavoured to make it appear that Mrs. Cumming's conduct was so irrational in regard to her children as to amount to a delusion; and it had been represented throughout the whole case, that her children had behaved towards her with uniform kindness; he apprehended if he could show, which he could do, that her children took part in this transaction, it was important evidence. Sir F. THESIGER replied that it could only be evidence if proved to have been communicated to Mrs. Cumming. The Commissioner decided that the evidence must be confined to what took place in Mrs. Cumming's presence, or to what was communicated to her. The evidence of this witness was consequently excluded.)

*James Oldfield*, examined.—A clerk at the London Monetary Company. Lives at 6, Edgeware-road; has lived there all his life. Saw Mrs. Cumming in the beginning of 1851, at Upper Stamford-street. I went there to get bail for Mrs. Cumming on this charge of perjury. I found two policemen there, and Ebenezer Jones came in. I was there when the police forced open the door. I heard Mrs. Cumming scream.

(The COMMISSIONER here addressed Dr. Hale upon the subject of a letter which he had received, signed by the medical witnesses, complaining of their exclusion from the court. The COMMISSIONER stated that they were sent out of court by those who subpœnaed them. He was quite sure they meant no disrespect. He had no jurisdiction. He said it was the practice for all witnesses to be excluded, except by consent. He referred to the *Times* of Saturday, where there was a discussion upon this question. He did so because he was aware that medical gentlemen might feel their exclusion not quite courteous. Dr. HALE observed that several medical gentlemen were under that impression. Sir F. THESIGER disavowed any intention of offering any slight, and repeated his argument, which has been already stated. Dr. HALE stated that medical gentlemen in the habit of attending Commissions believed this was the first time such a thing had been insisted on. The COMMISSIONER said he had himself given that excuse for what was stated.)

The witness's examination was resumed.—The police remained in the house some hours. The next day I attended to give bail at the police court. Mrs. Hickey was there, and Ebenezer Jones; they left the court together. A day or two after that I saw Mrs. Cumming with Mr. Haynes. She talked of changing her residence. She came to my house, and remained from March till June. I came home one day and found Mrs. Ince there. Mr. Haynes was there; he sent for me to witness what passed. I heard Mrs. Ince complaining of Mr. Haynes for keeping her away from her mother. Mr. Haynes said he was there by Mrs. Cumming's own desire. Mrs. Cumming said it was so. She complained of Mrs. Ince placing her in York House. Mrs. Ince was very much excited. She was in a passion. When Mrs. Cumming complained of her having put her in York House, Mrs. Ince said, "Mr. Haynes placed you there, mamma, and we got you out." Two or three days after that I remember Mrs. Ince calling again to see her mother. I was not present. The day after this, when I came home at half-past four, Mrs. Ince and Mrs. Hooper were at the door with a policeman, and two or three men. They said something to the policeman. They declined to interfere. They stayed after that some time at the door. During the time Mrs. Cumming was there she behaved like a sane person and a lady.

Cross-examined.—None of Mrs. Cumming's furniture was brought to the Monetary Loan Office. I never spoke to Mrs. Cumming about her daughters.

By the COMMISSIONER.—When Mrs. Ince was in the room with Mrs. Cumming there were no meals going on.

*Mr. Charles Ellis*, examined.—Lives at 7, North-street, Brighton. Was introduced to Mr. Haynes by Messrs. Webb, the auctioneers, of Brighton, on the 28th October last. I was instructed to go to the residence of Mrs. Cumming, at Bloomsbury-place. I saw a man and woman there who, I was informed, were keepers of a lunatic asylum. I went up into the drawing-room. Dr. King came about twelve o'clock. I asked him what his business was. He said he came to see Mrs. Cumming. I told him she was dozing. He went to Mrs. Cumming's bed-room, and called to Mrs. Watson (Mrs. Cumming's servant) to let him in. He called out to Mrs. Cumming—" Mrs. Cumming, I want to see you. I must come in, I am Dr.

King. I want to prescribe for you." When I asked him not to disturb Mrs. Cumming, he said he was Mrs. Cumming's medical-attendant; he said, "I have as much right here, in this instance, as the Lord Chancellor himself." I said, "Perhaps you will have the kindness to produce your authority;" he said, he had left it at home. He knocked two or three times after that, and I stopped him. In the evening of the same day I remember Mr. Turner being there. Mrs. Cumming had instructed me to keep the door locked and admit no one. Mr. Turner said I had refused to admit Dr. King to see her. Mr. Turner said we should all of us get sent to gaol, or something of that sort. The following day I recollect Dr. King coming with Mr. Chase, and several other men. They went up stairs to Mrs. Cumming's bedroom. I asked them their pleasure. Dr. King said they were come to remove Mrs. Cumming. He produced a paper which he read hurriedly. He demanded admittance into Mrs. Cumming's room. I heard her say—"Don't let them in." I saw Mr. Chase, the police-officer, break open the door. Dr. King gave directions. Mrs. Cumming screamed out "Oh! oh!" several times. She was very much alarmed. She said it was a very treacherous scheme, and wanted to know where they were going to take her to. They said they were about taking her away by a railway train. She called out again after that. She spoke to Dr. King in consequence of his remaining in the room while they were finishing dressing her. Dr King hurried the women very much. There was a fly at the door. Dr. King hurried the parties very much (in getting her into the fly); he said, "What are you about, we want you to make haste. Did you never get a woman into a fly?" and he put his arm on Mrs. Cumming, and she went in in some sort of style. She called out, "You have hurt me!" With the exception of the disturbance created by these parties in moving Mrs. Cumming, the house was peaceable and well conducted. I made no resistance.

Cross-examined.—I had verbal instructions from Mr. Webb. I did not see Mr. Haynes until after I left the house and came to London.

*Albina Watson*, examined.—I reside with Mrs. Cumming. I went as waiting-maid last year, in July; have been with her since. On the 27th October, I remember Sir A. Morison, Mrs. Turner, Mrs. Ince, and two policemen coming. The street door was opened to them by a girl named Sally Dunford. Mrs. Cumming had been in Brighton nearly a month. She used to drive out most days. She was a lady of cleanly habits. I never saw Mr. Haynes at Brighton till the day the house was entered. Mrs. Cumming told me she had invited him to dine there that day. When I saw them in the passage I ran up stairs and told Mrs. Cumming there were four gentlemen and a female, who, I thought, was her daughter. She desired me to lock the door. Mrs. Ince came to the door. She tried it. Mrs. Cumming said, "Who is there?" she said, "It is me, mamma." Mrs. Cumming said, "Catherine Ince, I wonder you dare to come to my house. You are no daughter of mine; I don't wish to see you." Mrs. Ince said to me, "Woman, I desire you to open the door." Mrs. Cumming said, "I desire her not; she is my servant, and I expect she will obey me." After Mr. Haynes came, some one asked to have the door opened, and told me if I did not have the door opened I should be liable to be sent to Newgate. Mr. Chase opened the door. Mr. Turner entered with Sir A. Morison. Mrs. Cumming asked for Mr. Haynes to be present, but Mr. Turner would not allow me to ask him. Sir A. Morison made inquiries as to the state of her health. He asked her whether she was unhappy or uncomfortable. She said she was not. He asked her if she had daughters; and why she would not see them. She said that she had no wish to see them; they had not treated her as daughters. Mr. Turner put several questions about her property. She objected to answer. During the whole time of the interview I can take upon myself to say that Mrs. Cumming did not say one word about her cats being her postillion and coachman. Mr. Turner took the most active part in examining Mrs. Cumming. He suggested a great many questions that were put by the physicians. He asked her a good many questions about Mr. Haynes. With the exception of my keeping the door shut at Mrs. Cumming's commands, I heard of no obstruction to the parties at all. I think Sir A. Morison's examination lasted an hour. At that time she was very unwell. Dr. King examined her, after Sir A. Morison was gone, for rather longer. Mr. Turner was present the whole time. [The witness then recounted Dr. King's call on the following day.] No attempt and no suggestion to remove Mrs. Cumming was made. She could not have been removed without great difficulty. The woman,

Ann Haines (keeper from the asylum), had not seen Mrs. Cumming until the day she was taken away. [The witness then related the circumstances of Mrs. Cumming's removal by Dr. King, Mr. Chase, &c., as given by last witness.] Mrs. Cumming's health had suffered since the first visit. While Dr. King was in her room, and she was dressing, she said to him, "For decency's sake, if you are a gentleman, leave the room." After that he came in again to hasten the persons who were dressing her. She screamed as she was forced into the fly. I requested permission to accompany her. Mrs. Cumming requested me to do so. Dr. King said I was not to go. She had given me her money before this, 40*l.*; she expected me to go with her. During all the time I have been with Mrs. Cumming she has behaved reasonably. She is of an irritable temper. Mrs. Cumming resumed her name when she got to Brighton.

Cross-examined.—I heard Sir A. Morison's examination. I did not hear her say Mrs. Ince had tried to strangle her at Howley Villa. She said that she came to see her, and put her arms about her, and she thought at the time it was an *attempt* to strangle her. She said, after the treatment she had received she did not think it was from a kind feeling. I will swear that she did not say to Sir A. Morison that they had tried to murder her. I will swear she did not say to Sir A. Morison that her daughters wished to poison her, and had put poison into her tea-cup. I did not hear her say to Dr. King she had a great hatred of her daughters because they had attempted to poison or strangle her.—Q. Did you hear her speak to Dr. King about her late husband? A. Those questions were put by Mr. Turner. She was asked if she thought it was her daughters who put poison in the milk. She said, no, she did not. It was Mr. Turner asked her that. She said she did not know who put it there.

Re-examined.—Mr. Turner asked her about her seeing her husband improperly acting with the servant. He asked her if she did not catch Captain Cumming with the servant. She did not answer him. She said he was dead and gone, and that his faults must rest with him; and she thought it mean and pitiful for anyone to revive those things.

*Mrs. Mary Moore*, examined.—Mr. Parkin, the former proprietor of York House Asylum, is an old friend. He introduced me to Mrs. Cumming when she was at the asylum in 1846. I dined then with Mr. Parkin and Miss Parkin. She called upon me after her liberation. I frequently saw her at Camberwell. In 1848, I went to reside at St. John's Wood. From that time I have been in the habit of seeing her. I have been with her shopping. She demeaned herself with shrewdness and propriety; always as a lady, and they have acted so to her. In the month of November last, I found I was appointed by the Court of Chancery to reside with Mrs. Cumming. Up to her late seizure, her state of mind and conduct were similar to what I had before observed. I should not have visited her if I had seen anything different. I found her a lady. Her memory weakens. I remember Dr. Aldis calling on Mrs. Cumming. It is quite false that I held up my finger to Mrs. Cumming, or made any signs to her. I made no effort to prevent her speaking. Mrs. Cumming never spoke. I never observed anything offensive from the cats; if I had, I should not have gone into Mrs. Cumming's house. Since her discharge from Effra Hall she has appeared to me to be greatly affected physically. She is much weaker than when I knew her before. She has been very much exhausted after seeing different medical men, and after being seen by the jury.

Cross-examined.—I did not make any signs when Dr. Davey visited Mrs. Cumming. I do not think Mrs. Cumming at all likely to be influenced by signs made by those she knows. She is quite capable of judging, and (has) a very keen judgment. I never asked her about her will or her affairs.

Re-examined.—My husband was a physician. I treated Mrs. Cumming as other ladies.

By the Commissioner.—I think that since her visit to Effra Hall her memory is weaker than it was, but she has a perfectly sound understanding on all subjects. I do not know that she takes any particular quantity of wine—no spirits. I never had any conversation with her about her daughters. I am not in the least afraid of exciting her about them.

*Mr. Jacob Hibbert*, examined.—A builder in St. John's Wood. (This witness knows the houses bought by Mrs. Cumming in the Queen's-road, and says he would not build them for less than 1600*l.*)

*Mr. Frederick Lomax.*—Auctioneer and surveyor. Witness's valuation of the houses in 1847 was 1610*l.*, that would be 7*l.* per cent., deducting ground rent and insurance. On re-examination, witness said the property in that neighbourhood had improved since he had valued the houses.

*Mr. William Wright Lucking*, examined.—Auctioneer and surveyor. The houses would fetch 1650*l.* to-morrow.

*Mr. George Chadwin.*—Vestry clerk of Battersea. Produced the original order concerning the order of affiliation upon Captain Cumming, on the 21st September, 1822.

